# New photic stimulating system with white light-emitting diodes to elicit electroretinograms from zebrafish larvae

**DOI:** 10.1007/s10633-017-9602-1

**Published:** 2017-07-29

**Authors:** Hisashi Matsubara, Yoshitsugu Matsui, Ryohei Miyata, Yuhei Nishimura, Tetsuro Yamamoto, Toshio Tanaka, Mineo Kondo

**Affiliations:** 1 Department of Ophthalmology, Graduate School of Medicine, Mie University, 2-174 Edobashi, Tsu-city, Mie 514-8507 Japan; 2 Department of Molecular and Cellular Pharmacology, Pharmacogenomics and Pharmacoinformatics, Graduate School of Medicine, Mie University, 2-174 Edobashi, Tsu-city, Mie 514-8507 Japan; 3 Department of Neurophysiology, Graduate School of Medicine, Mie University, 2-174 Edobashi, Tsu-city, Mie 514-8507 Japan; 4 Department of Systems Pharmacology, Graduate School of Medicine, Mie University, 2-174 Edobashi, Tsu-city, Mie 514-8507 Japan

**Keywords:** Zebrafish, Electroretinography (ERG), Light-emitting diodes (LED), Light stimulator, Visual system, Retina

## Abstract

**Purpose:**

The zebrafish is an established animal model commonly used in biological, neuroscience, and genetic research. We have developed a new light stimulating system using white light-emitting diodes (LEDs) to elicit ERGs from zebrafish larvae. The purpose of this study was to record full-field ERGs and to evaluate the inter-trial reliability of the ERGs recorded with our system from zebrafish larvae.

**Methods:**

The stimulating device used white LEDs that were attached to a stereomicroscope, and the location of the recording electrode on the cornea could be monitored while the eye was being stimulated. Full-field scotopic and photopic ERGs were recorded from larvae at the age of 5–7 days post-fertilization (dpf). Intensity–response curves were constructed from the ERGs. Inter-trial reliability of the ERGs recorded by our system was evaluated.

**Results:**

This stimulating system could be used for efficient and reliable ERG recordings from 5–7 dpf larvae. The amplitudes, implicit times, and the waveforms of the scotopic and photopic ERGs were similar to those reported in earlier studies. Inter-trial reliability of the amplitudes of the photopic ERG b-waves was excellent with an intra-class correlation coefficient of 0.98.

**Conclusion:**

We conclude that this new light stimulation system using white LEDs attached to a stereomicroscope will be helpful in recording reliable ERGs from zebrafish larvae.

## Introduction

The zebrafish is a well-established and useful animal model for biological, neuroscience, and genetic studies of development because they are small, easy to breed, translucent, and inexpensive [1]. For studies on the visual system, different genetic varieties and evaluation methods have been used to assess the properties of the visual system [2–5]. The procedures that have been widely used to examine the responses elicited by visual stimuli include the optokinetic reflex (OKR), optomotor response (OMR), visual startle response, and prey-capture response [6–11].

Electroretinography (ERG) has been used to evaluate the physiological properties of the retina of many different animals including humans, and the ERGs that are elicited by light stimulation represent the electrical potential changes in the different neurons in the retina [12]. The ERGs are made up of different components that originate from specific retinal neurons, and alterations on one component of the ERG can offer a clue on the specific type of retinal neuron that has been altered.

In previous zebrafish studies using ERGs to assess the physiology of the retina, the light stimulation methods varied, and many authors have used light mainly from a fiber optic system that was placed in front of the eye that is being evaluated [6, 13–17]. Because the intensity of the light on the retina and the illuminated retinal area can easily change by the position of the zebrafish relative to the fiber optic bundle, this variability can affect the results.

One way to reduce the stimulus variability would be to stimulate the entire retina uniformly, i.e., ganzfeld stimulation. The ganzfeld stimulation method is an established technique that stimulates the entire retina uniformly. For this, the eye is placed in a ganzfeld bowl, and the light stimulus is reflected from the inner surface of the bowl and stimulates the entire retina. This ganzfeld technique has been used to elicit reliable ERGs from zebrafish larvae [18, 19]. The investigators first set the larvae on a recording table, and a glass microelectrode is positioned on the center of the cornea while viewing the preparation with a stereomicroscope. The recording table is then placed into the ganzfeld bowl [18] or covered by the ganzfeld bowl [19]. Because these methods require carrying the recording table into the ganzfeld bowl or covering the recording table by the ganzfeld bowl, there is a possibility that the tip of the microelectrode could slip off from the cornea during the time of transit. This makes it difficult to confirm the location of the microelectrode since the larva has been placed in the ganzfeld bowl.

To simplify the setup and illuminate the retina uniformly, we constructed a new light stimulator that can be attached to a stereomicroscope. The source of the light stimuli was white light-emitting diodes (LEDs). The aim of this study was to determine whether this new light stimulus system can elicit reliable and reproducible ERGs from zebrafish larvae.

## Subjects and methods

### Animals and ethics statement

The embryos of zebrafish (*Danio rerio*) of the RIKEN wild-type strain (RIKEN WT) were obtained from the National BioResource Project of the RIKEN Brain Science Institute (RIKEN, Saitama, Japan). The embryos and larvae were maintained under a 14-hour light (approximately 500 lx) and 10-hour dark cycle, and the temperature of the aquarium E3 medium (5 mM NaCl, 0.17 mM KCl, 0.33 mM CaCl_2_, and 0.33 mM MgSO_4_) was 28 °C. The age of the larvae was based on the days post-fertilization (dpf), and they were tested at an age of 5–7 dpf, which is the age that has been commonly used in studies on the visual system of zebrafish larvae. All experiments were performed at room temperature (28–30 °C) and in the afternoon. After the completion of the experiments, the larvae were euthanized by a lethal dose of 3-aminobenzoic acid methyl ester (MESAB; Sigma, St. Louis, MO, USA).

The research was conducted in full compliance and strict accordance with the Association for Research in Vision and Ophthalmology (ARVO) Resolution on the Use of Animals in Ophthalmic and Vision Research. The protocol was approved by Mie University Graduate School of Medicine Institutional Animal Care and Use Committee.

### LED light stimulator

**Fig. 1:**
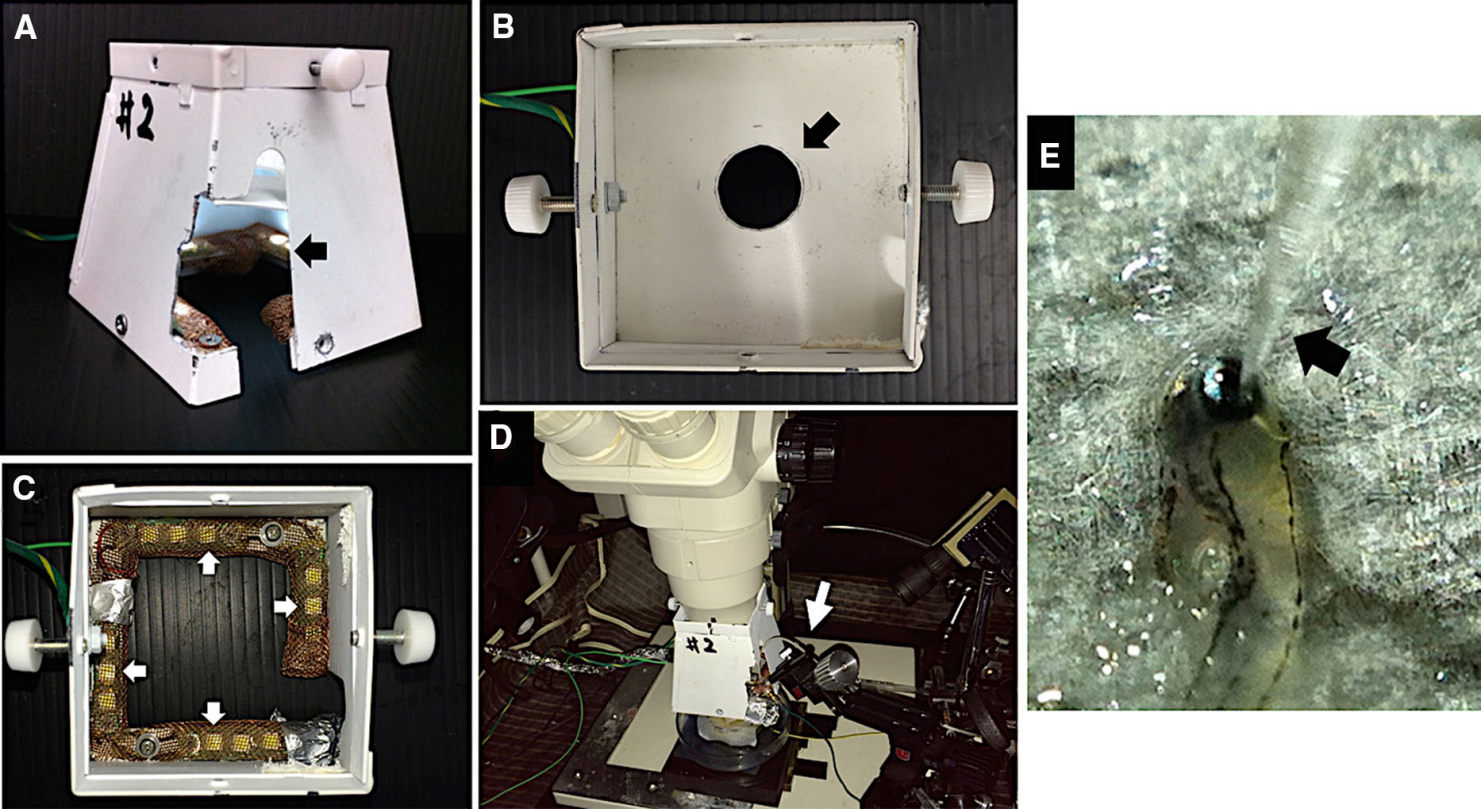
A built-in light-emitting diode (LED) light stimulator. **a** External view and **b** internal view from the upper part of the LED light stimulator. The partition plate has a circular hole of 2.0 cm in diameter (*arrow*). **c** The inside of the partition plate is removed. Twelve white LEDs are installed in the lower part of the stimulator (*arrows*). All LEDs are covered with copper netting. **d** The stimulator can be attached to the lower part of a stereomicroscope. **e** The view of larvae and a glass microelectrode (*arrow*) with a stereomicroscope. The damp paper towel which covered the larva’s body is removed

Our custom-made LED light stimulator (MAYO Co., Inazawa, Japan) was constructed in a quadrangular shape (Fig. 1a). The dimensions of the quadrangle were 8.0 cm wide, 8.0 cm deep, and 8.0 cm high. The bottom and top surfaces were open. A part of the right wall was cut so that the glass microelectrode could be mounted on the microelectrode holder located in the quadrangular light stimulator (Fig. 1a, arrow). The inside of the quadrangle was partitioned off with a plate which had a circular hole of 2.0 cm in diameter (Fig. 1b, arrow). After painting the inside of the quadrangle and partition matte white, twelve white LEDs (NS6W083BT, NICHIA Corporation, Tokushima, Japan) were installed on the inside of the lower part of the quadrangle (Fig. 1c, arrows). These LEDs served as the source for the stimulus and the background illumination.

All LEDs were covered with copper nets to reduce the electrical artifacts (Fig. 1c). The LEDs were driven by an electronic control unit (LS-100, MAYO Co., Inazawa, Japan), which controlled the light intensity (current) and duration of the stimulus. Light from the LEDs was reflected from the inner surface of the enclosure. This setup was designed so that it could be attached to the lower part of a stereo or dissecting microscope without blocking the view of the larvae (Fig. 1d).

### Recording platform and set up

**Fig. 2:**
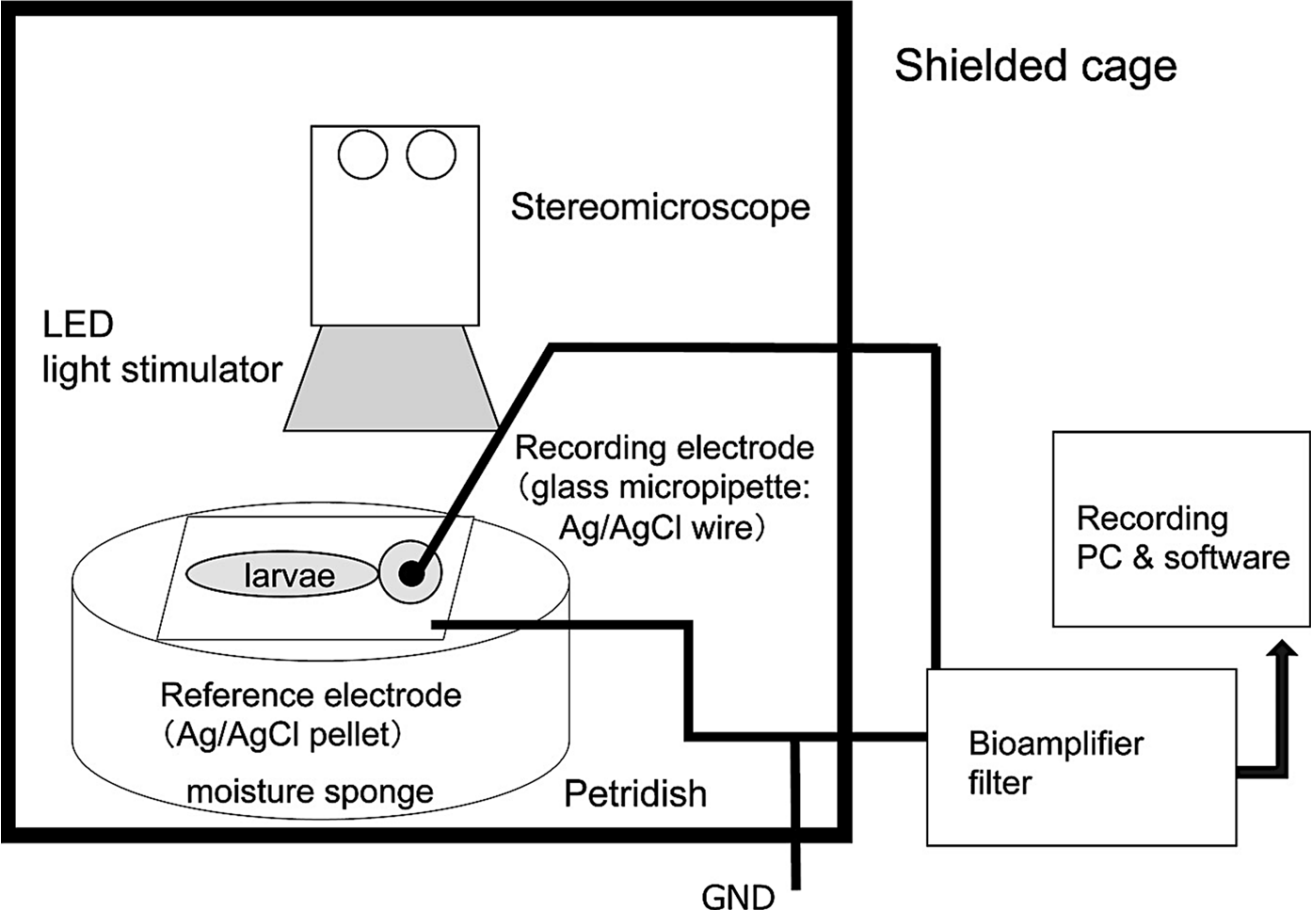
Diagram of ERG recording setup

All of the electrodes, stereomicroscope, and the LED light stimulator were placed inside a grounded shielded cage of 80 × 70 × 140 cm (Fig. 2). In our system, an anti-vibration table was not used to improve the signal-to-noise ratio [19].

After filling a glass micropipette with an opening of about 20 μm at the tip with E3 medium, a chloride silver wire electrode was inserted the micropipette and then fixed to a microelectrode holder (E45SW-F10PH, Warner Instruments, Hamden, CT, USA). The glass microelectrode holder was fixed and moved by a micromanipulator (UM-1PF: Narishige Group, Tokyo, Japan, Fig. 1d, arrow) so that the tip of the microelectrode was correctly placed on the center of the cornea using a stereomicroscope. The reference electrode was a chlorided silver pellet that was placed under a moist paper towel that was resting on a sponge in a 35 mm Petri dish containing E3 medium.

The chlorided silver wire in the microelectrode and the reference electrode were connected to a bioamplifier (AVB-10, Nihon Kohden, Tokyo, Japan). The electrical signals from the larva were differentially amplified 2000 times with bandpass cut-off frequencies of 0.8 and 300 Hz for all of the recordings. The amplified signals were fed to a PowerLab 2/25 instrument (ADInstruments Pty. Ltd., South Wales, Australia) using the Scope version 4.1 software (AD Instruments Pty. Ltd.), and data acquisition, storage, and analyses were performed with a personal computer (iMac^®^; Apple Computer, Inc., Cupertino, CA, USA).

### Experimental procedures

ERGs were recorded under scotopic (dark-adapted) and photopic (light-adapted) conditions. All specimens were dark-adapted for 30–40 min prior to the recordings, even though a shorter period of dark adaptation is reported to be sufficient in 5–7 dpf larvae [19]. All of the preparations and setups were done under dim red illumination to minimize light adaptation. After the dark adaptation, the larvae were anesthetized by submersion in a solution of 0.02% MESAB in E3 medium until the swimming motions stopped. They were then paralyzed by submersion in a 0.8 mg/ml Esmeron (Organon Teknika, Eppelheim, Germany) solution in E3 medium. They were then positioned on their side on a piece of moistened paper towel that was placed on a reference electrode in a Petri dish (Fig. 2.). Larvae’s entire body except the head was covered with a small strip of paper towel moistened with a solution of 0.02% MESAB in E3 medium. The damp paper towel kept the larvae moist during the experiments. Then, the Petri dish was placed on the stage of the microscope, and the glass microelectrode was positioned at approximately the center of the cornea. Although we did not supply the O_2_ during the ERG recording, the experimental period was less than 30 min.

### Electroretinography

The electroretinograms (ERGs) were recorded under scotopic (dark-adapted) and photopic (light-adapted) conditions. After the larvae were positioned under the stereomicroscope under dime red illumination, they were dark-adapted additionally for more than three minutes in complete darkness prior to the scotopic ERG recording. For the photopic ERG recordings, the larvae were light-adapted to a background illumination (31.6 cd/m^2^) for over 5 min before the recordings. The stimulus intensity was changed in 0.5 log unit steps from −3.0 log units to 0 log units with log 0 corresponding to 3160 cd/m^2^ (photopic unit). The background luminance was 31.6 cd/m^2^ for photopic condition which was similar to that used in earlier studies [18]. The stimulus duration was 1000 ms which allowed the recordings of the ON and OFF responses separately. For each stimulus intensity, 3–10 responses were averaged with an inter-stimulus interval of 10–60 s for scotopic conditions or 3 s for photopic conditions.

The amplitude of the b-wave was measured from the trough of the a-wave to the peak of the positive b-wave. The implicit times of the b-wave were measured from the stimulus onset to the peak of the b-wave.

### Set up reliability test

To evaluate the reliability of our recording conditions, we determined the variations in the b-wave amplitude among the larvae under the same conditions. These trials consisted of placing the glass electrode on the cornea and recording the ERGs three times under the same conditions from 12 larvae. The stimulus intensity was −1.0 log unit on a steady background of 31.6 cd/m^2^.

### Statistics

Two-way layout ANOVA was used to evaluate the variations of the inter-recording setup trials and the inter-individual differences. To evaluate the reliability of the inter-recording trials, intra-class correlation coefficients (ICC) were also calculated. The ICCs were classified as: ‘excellent’ (≧.81), ‘good’ (.61–.80), ‘moderate’ (.41–.60), and ‘poor’ (≦.40) according to past biometrical studies [20, 21]. All statistical analyses were performed with the SPSS Statistics version 23. A *P* value of <0.05 was considered significant.

## Results

### System operability

We were able to adjust the position of the larvae and place the glass electrode at approximately the center of the cornea easily and quickly. We were also able to confirm and adjust the position of the microelectrode at any time during the experiment. It took approximately one to two minutes for the set up and not more than 30 min for recording all scotopic and photopic ERGs.

### ERG waveforms and intensity–response curve

**Fig. 3:**
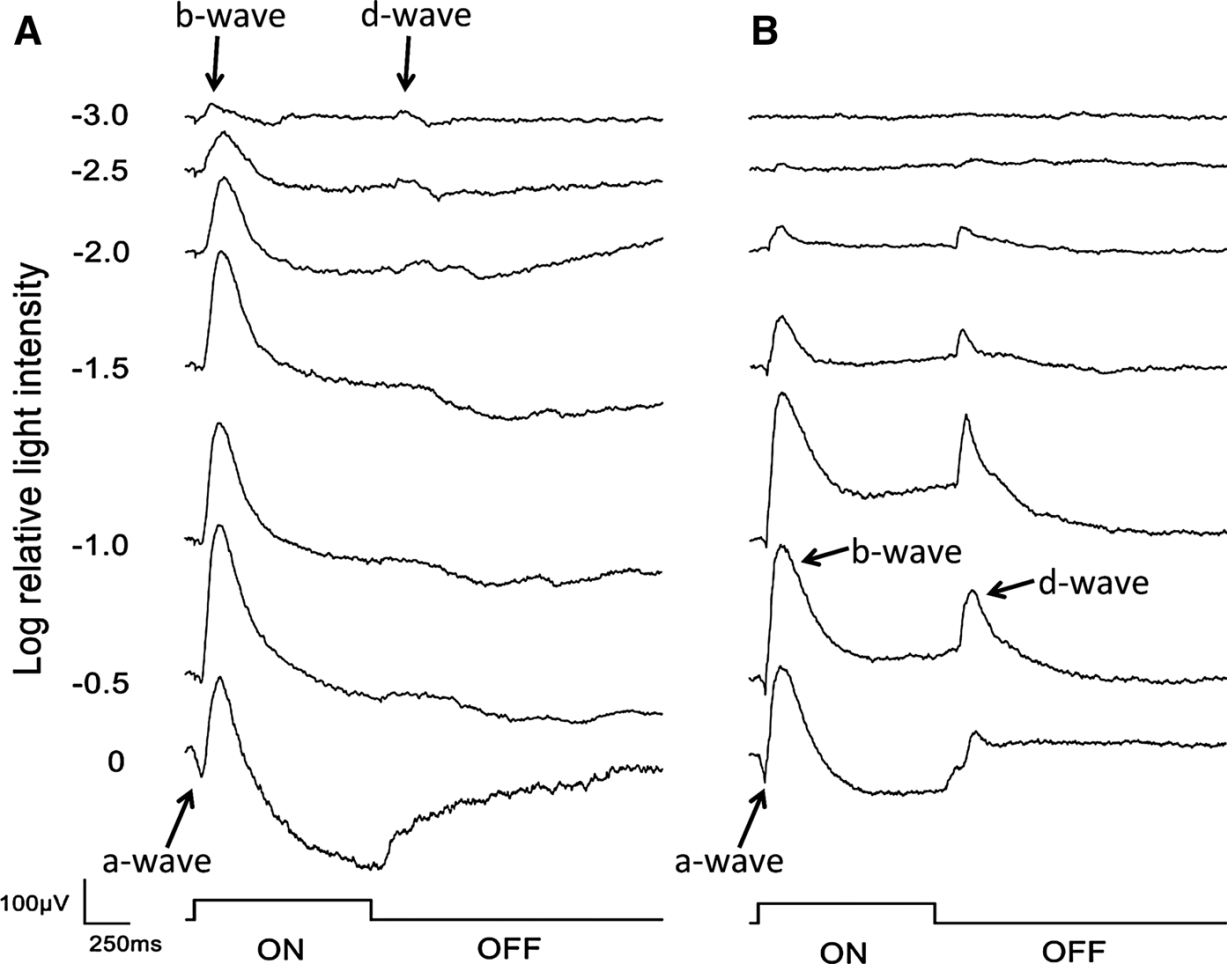
Representative ERG responses recorded by a 1000 ms stimulus. The intensity series was recorded under scotopic (dark-adapted, **a**) and photopic (light-adapted, **b**) conditions from 6 dpf zebrafish larvae. The stimulus intensities are −3.0, −2.5, −2.0, −1.5, −1.0, −0.5, and 0 log units. The stimulus intensity, log 0, corresponds to 3160 cd/m^2^ (photopic unit)

The a-, b-, and d-waves were recorded under both scotopic and photopic conditions, and their shapes were similar to those reported earlier for zebrafish larvae [6, 13–19]. Under scotopic conditions (Fig. 3a), positive b- and d-waves were present at the lowest stimulus intensity of −3.0 log units tested. The a-waves were very small and were first observed at −1.5 to −1.0 log units. As the intensity increased, the b-wave gradually increased while the positive d-waves were not clearly observed at the middle intensities of −1.5 to −1.0 log units. At the maximum 0 log unit intensity, the a-wave and a large negative-going wave after the b-wave were present while the positive-going d-wave appeared again.

Under the photopic conditions (Fig. 3b), the b- and d-waves were not observed at the lowest intensity (−3 log units) but were present at −2.5 log units. The amplitudes of both increased with the increase in stimulus intensities. The a-wave was observed at −1.5 to −1.0 log unit and increased with the increases in the stimulus intensities.

**Fig. 4:**
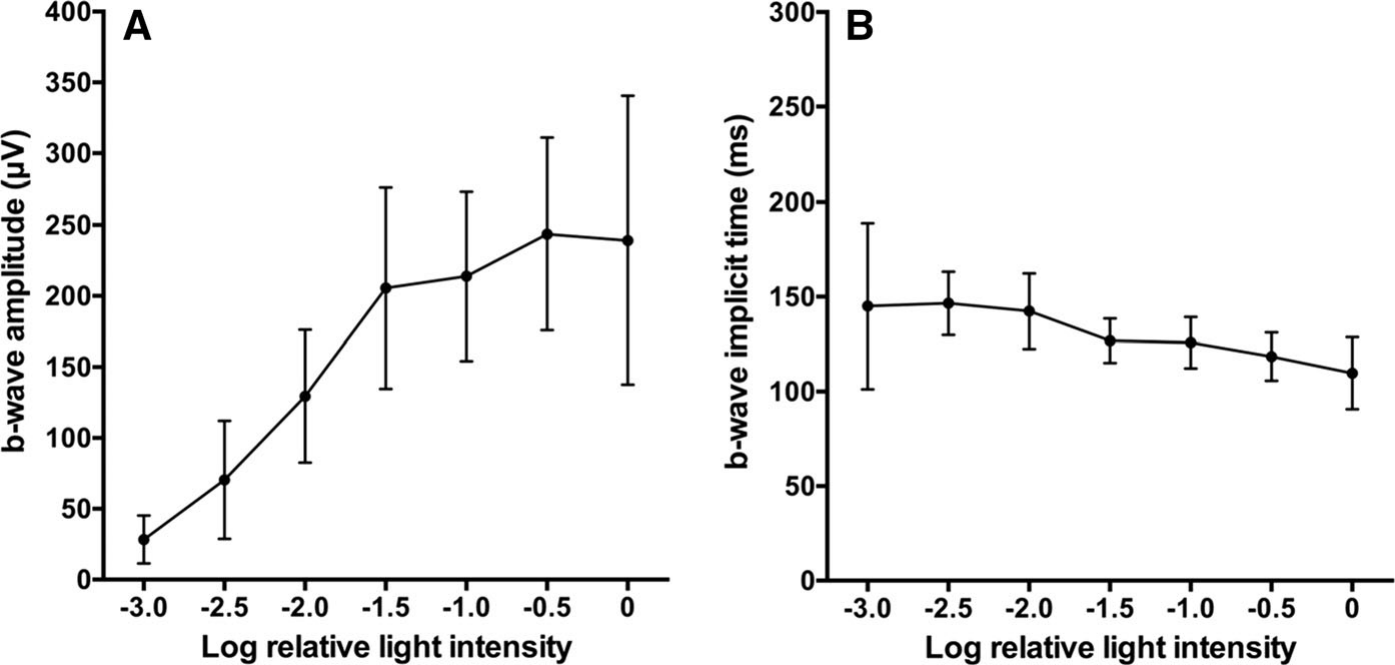
Plots of amplitude (**a**) and implicit time (**b**) as a function of light stimulus intensity under scotopic conditions. *Error bars* are 95% CI. The data from 12 zebrafish larvae are used

The changes in the amplitudes and implicit times of the scotopic ERG b-waves are plotted as a function of stimulus intensity in Fig. 4a, b, respectively (*n* = 12). The amplitudes of the scotopic ERG b-wave increased rapidly as the stimulus intensity increased at the lower stimulus intensities of −3.0 to −1.5 log unit; then, it increased more slowly until it plateaued at −0.5 to 0 log unit. In contrast, the scotopic ERG b-wave implicit times decreased monotonically with the increase in stimulus intensity.

### Set up reliability evaluations

**Fig. 5:**
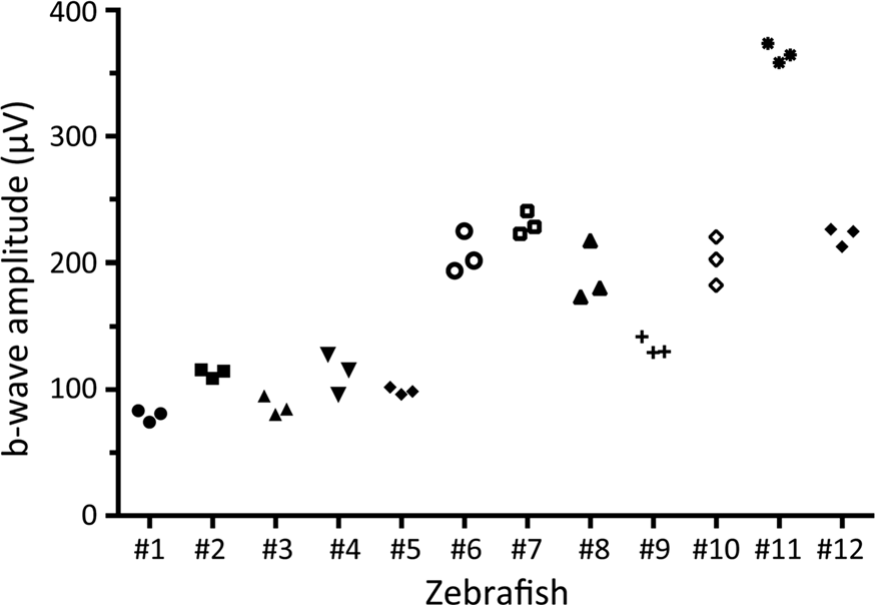
Plots the b-wave amplitudes of photopic ERG, in which the mounting the glass electrode on the cornea and recording the ERGs were repeated three times under the same conditions from 12 larvae

The amplitudes of the b-wave amplitudes of the photopic ERG in which placing the glass recording electrode on the cornea and recording the ERGs were repeated three times under the same conditions from 12 larvae are plotted in Fig. 5. The differences in the b-wave amplitudes for the inter-recording trials were not significantly different (167.1 ± 85.3 μV, 169.9 ± 79.0 μV, 173.2 ± 85.5 μV; *F* = 0.71; *P* = 0.50; two-way layout ANOVA). However, the inter-individual differences were statistically significant (*n* = 12 larvae, *F* = 134.9; *P* < 0.001; two-way layout ANOVA). The inter-trial reliability (intra-class correlation coefficient, ICC) was 0.98 for the three trials. These results indicated that the inter-trial reliability of the ERG recorded by our system was ‘excellent,’ and the variability in the b-wave amplitudes was mainly caused by the differences between the subjects.

## Discussion

Our results demonstrated that the waveforms of the ERGs recorded with our system (Fig. 3) were similar to those reported using fiber optic stimulating systems [6, 13–17] or a ganzfeld system [18, 19]. The amplitudes and implicit times were also comparable to that reported [6, 13–19]. We found that the inter-trial reliability was excellent at 0.98 (Fig. 5) in our recording system.

The diameter of the cornea of 5–7 dpf larvae is approximately 200 μm. Therefore, it is difficult to position the tip correctly at the center of the cornea with the naked eye and for the tip to make good electrical contact with the cornea to reduce the noise. Thus, performing these operations under a stereomicroscope is essential which is possible with our photic stimulation system.

The reason why such a high inter-trial reliability was obtained by our recording system is that the light stimulator is attached to the lower part of a stereomicroscope. With this device, the ERG recordings can be begun immediately after the tip of the microelectrode is placed on the center of the cornea while viewing the eye with a stereomicroscope. Thus, our device avoids the displacement of the tip of the microelectrode off of the center of the cornea during the setup. This is the greatest advantage in our ERG recording system for zebrafish larvae.

We used white LEDs as a light source because they are compact and cause less increase in the surrounding temperature when they are on, and it is easy to change their luminance. Although we tried to cover the LEDs with copper nets to reduce the electrical artifacts, we were not able to eliminate the electrical artifact completely (Fig. 3). The electrical artifact is specific and cannot be avoided when using LEDs to some extent. However, because these artifacts were very small and occurred at approximately 10–20 ms preceding the a-wave and at 100 ms preceding the d-waves, these artifacts should not influence our results.

For our photic stimulus system, we used a quadrangular box for the stimulating system (Fig. 1), and the white light from the LEDs is reflected from the inner surface of the box and stimulates the entire retina. However, the dome shape would be better to stimulate the entire retina evenly. In addition, our stimulator has a gap of about 1 cm from the eye level of the larvae to the lower edge of the stimulator. This gap made it possible to place and adjust the reference electrode, but the stimulation light from the horizontal and lower areas is not directed to the eye. Based on these reason, one question still remain as to whether our photic stimulating device stimulates the entire retina of zebrafish. Thus, this problem can be fixed by changing the shape and location of the stimulator in the next step.

There are three limitations in this study. The first limitation is that although we demonstrated an ‘excellent’ inter-trial reliability (ICC, 0.98), we did not compare the values of ICC between our system using full-field stimulus system and conventional zebrafish ERG system using a fiber optic directly. We are planning such direct comparative experiments when a next improved version of stimulator is developed.

The second limitation is that the evaluated intensities of stimulation were from −3.0 to 0 log units and the duration was 1000 ms only. Under scotopic condition, the b- and d-waves were already present at the lowest intensity of −3.0 log units. Therefore, to evaluate retinal functions in more detail, e.g., the scotopic threshold response, we need to evaluate the ERG responses at lower intensities.

The third limitation is that we evaluated only larvae of 5–7 dpf. Because it is known that there are differences in retinal function between larvae and adult zebrafish [13, 14, 22, 23], it is necessary to investigate whether our ERG system is also useful in evaluating ERGs from adult zebrafish.

In summary, we have developed a new light stimulating device for ERG recordings of zebrafish, in which the stimulator is attached to a stereomicroscope to reduce the steps of setting up the larvae for light stimulation. The results showed that our system had excellent inter-trial reliability, and therefore, it can be used for ERG recordings in zebrafish larvae.
